# Microbiota and Its Impact on the Immune System in COVID-19—A Narrative Review

**DOI:** 10.3390/jcm10194537

**Published:** 2021-09-30

**Authors:** Marzena Jabczyk, Justyna Nowak, Bartosz Hudzik, Barbara Zubelewicz-Szkodzińska

**Affiliations:** 1 Department of Nutrition-Related Disease Prevention, Faculty of Health Sciences in Bytom, Medical University of Silesia, Piekarska 18 Street, 41-902 Bytom, Poland; marzena.jabczyk@gmail.com (M.J.); bzubelewicz-szkodzinska@sum.edu.pl (B.Z.-S.); 2 Department of Cardiovascular Disease Prevention, Faculty of Health Sciences in Bytom, Medical University of Silesia, Piekarska 18 Street, 41-902 Bytom, Poland; bartekh@mp.pl; 3 Silesian Center for Heart Diseases, Third Department of Cardiology, Faculty of Medical Science in Zabrze, Medical University of Silesia, 41-800 Zabrze, Poland

**Keywords:** microbiota, pandemic, COVID-19, lungs microbiome, SARS-CoV-2

## Abstract

The microbiota is of interest for the development of a therapeutic strategy against SARS-CoV-2 coronavirus disease 2019 (COVID-19) due to its impact on the host immune system. Proven communications of the gut microbiota with the pulmonary microbiota (gut–lung axis) and the pathway of neural connections between the gut and brain (gut–brain axis) may be important in the face of the pandemic. SARS-CoV-2 was shown to affect almost all organs because of the presence of a host receptor known as angiotensin converting enzyme 2 (ACE2). The ACE2 receptor is mainly present in the brush border of intestinal enterocytes, ciliary cells, and type II alveolar epithelial cells in the lungs. The transport function of ACE2 has been linked to the ecology of gut microbes in the digestive tract, suggesting that COVID-19 may be related to the gut microbiota. The severity of COVID-19 may be associated with a number of comorbidities, such as hypertension, diabetes, obesity, and/or old age; therefore, attention is also paid to multiple morbidities and the modulation of microbiota through comorbidities and medications. This paper reviews the research in the context of the state of the intestinal microbiota and its impact on the cells of the immune system during the SARS-CoV-2 pandemic.

## 1. Introduction

The gut microbiota and its connection with the functioning of the innate and adaptive immune systems constitute an active area of scientific research [1]. Homeostasis of the immune system is achieved in part through a wide interaction between the gut microbiome and mucosal immune system [2]. Microorganisms present in the gut are a source of antigens that continually stimulate the intestinal lymphatic tissue, known as the gut-associated lymphoid tissue (GALT), and thus, the immune system [3]. Genetic data indicate that the human body contains only 1% of human genes, and 99% of the genes belong to the microorganisms that inhabit the host [4]; therefore, the gut microbiome of an individual can encode more than 150 times more genes than the host genome. In contrast to the host genome, the quantitative and qualitative composition of microbiota is dynamic and changes under the influence of environmental factors, including the diet, exposure to antibiotics and, in particular, in response to disease [5].

In the face of the COVID-19 pandemic caused by SARS-CoV-2 beta-coronavirus, it is essential to recognize the mechanisms responsible for the host immune response caused by contact with the virus. Due to frequent symptoms indicative of respiratory disease in patients infected with SARS-CoV-2, the lungs appear to be the primary organ affected by COVID-19 disease. However, the development of organ dysfunction during the course of the disease has been reported, such as dysfunction of the intestine or liver or multiple organ failure [6,7,8,9,10]. The occurrence of diarrhea in patients with COVID-19 and a high mortality rate in elderly patients indicate a possible interaction of the gut–lung axis during the course of COVID-19. The diversity of the gut microbiota decreases with age; thus, dysbiosis can be partially responsible for the high risk of severe illness caused by SARS-CoV-2 in older adults. Disruption of the integrity of the intestinal barrier due to dysbiosis can result in the translocation of the SARS-CoV-2 virus from the lungs into the intestinal lumen via the circulatory system and lymphatic vessels [6]. Improving the intestinal microflora system by personalized nutrition is thought to enhance immunity and may be a significant prophylactic factor that minimizes the impact of viral infections in the elderly and immunocompromised patients, particularly in the face of the COVID-19 pandemic [1].

The information on the gut–lung cross talk and possible associations of gut microbiome and COVID-19 is scarce. This review summarizes the available knowledge and possible links of intestinal microbiota with immune response to SARS-CoV-2 infection and COVID-19 severity.

## 2. Gut Microbiota in SARS-CoV-2 Infection

The gut microbiota is the largest reservoir of microorganisms (bacteria, archaea, viruses, and fungi) inhabiting the human body [1,5]. The intestinal bacteria in healthy subjects primarily consists of *Actinobacteria, Firmicutes, Proteobacteria*, and *Bacteroidetes* [11], while the colon harbors bacteria of the *Bacteroidaceae, Prevotellaceae, Rikenellaceae, Lachnospiraceae*, and *Rumniococcaceae* families [12]. The previously known functions of these microorganisms comprise the regulation of the physiological functions of the host, including metabolism of nutrients, digestive processes, and the regulation of the development and functioning of the innate and adaptive immune systems [1,13]. Furthermore, the commensal gut microbiota influence mitochondrial functions related to energy production, redox balance, mitochondrial biogenesis, and inflammatory cascades, enhancing the synthesis of metabolites, including short-chain fatty acids (SCFAs) and secondary bile acids. Viral or bacterial infections are stress factors to the organism and may induce the modulation of the immune response by mitochondria, which leads to inflammation. The mitochondria were reported to affect the microbial system by activating intestinal effector cells, including immune cells and epithelial enterochromaffin cells [14,15].

Groves et al. [16] analyzed the relationship between viral infections, food consumption, gut microbiome, and gut metabolome. The study showed that changes in the composition of the intestinal microflora after viral infection may be caused by multiple factors, including reduced food intake, which is also associated with the activity of CD8^+^ T cells and significant changes in the gut metabolome after lung infection that was reported to increase the levels of produced lipids. Current studies demonstrate that SARS-CoV-2 may affect almost any organ in the human body due to the presence of a receptor known as angiotensin converting enzyme 2 (ACE2) on multiple cell types [17,18,19,20]. This receptor is present in the brush border of intestinal enterocytes, ciliary cells, and type II alveolar epithelial cells in the lungs [18]. The function of amino-acid transport performed by ACE2 has been linked to the ecology of gut microbes in the gastrointestinal tract, suggesting that COVID-19 may be related to the gut microbiota to a degree [20].

It is possible that the small intestine plays a key role in the dynamics of COVID-19 development. There is evidence that ACE2 has been expressing in the stomach, ileum, colon, kidney, and liver even higher compared to the lungs [17]. Moreover, Lamers et al. [18], in their study, have demonstrated that SARS-CoV-2 attaches to ACE2 receptors on enterocytes. Binding to human ACE2 indicates that high levels of SARS-CoV-2 are present in the intestine, especially in colonocytes of healthy individuals, where the virus may regulate the transport of amino acids and the quantitative and qualitative composition of the microbiota and induce inflammation in the gut [17,21,22]. Furthermore, Zuo et al. [19] observed not only that some bacterial strains in the gut (*Bacteroides dorei, Bacteroides thetaiotaomicron, Bacteroides massiliensis*, and *Bacteroides ovatus*) that are correlated inversely with SARS-CoV-2 in fecal samples from patients have been decreasing in the expression of ACE2 in murine gut, but also, they have observed a correlation of the abundance of *Coprobacillus, Clostriudium ramosum*, and *Clostridium hathewayi* with COVID-19 severity [19].

The ACE2 enzyme and transmembrane serine protease 2 are expressed in a wide variety of human tissues, including, but not limited to, the esophagus, lung, liver, kidney, brain, colon, or small intestine epithelium. Blocking ACE2 leads to an increased level of angiotensinogen and, in consequence, may lead to hypertension [23]. SARS-CoV-2 activates intestinal ACE2 receptors and induces inflammation and gastrointestinal symptoms, such as diarrhea [20]. Microbiota dysbiosis caused by alteration in the T and B lymphocytes of the intestinal immune system and activation of the enteric system sends inflammatory mediator signals to the cardiovascular system or other human organs, including the brain [20].

## 3. The Microbiome–Gut–Brain Axis in SARS-CoV-2 Infection

**Figure 1:**
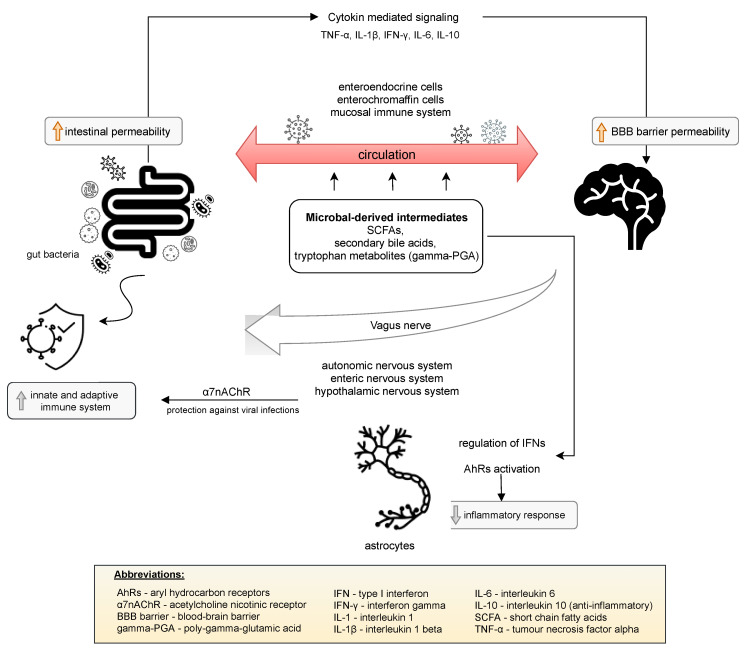
Bidirectional interaction of the gut–brain axis.

The presence of SARS-CoV-2 in the gastrointestinal tract may affect that virus entering the central nervous system (CNS) through the vagal nerve or the circulatory pathway [24]. The bidirectional communication network between the gut and the central nervous system (CNS) is defined as the gut–brain axis. The vagus nerve, enteric nervous system, autonomic nervous system, and hypothalamic pituitary adrenal axis are responsible for the transmission of information between the CNS and the gastrointestinal tract. Several mechanisms are involved in the interaction of the gut microbiota with the CNS. Haase et al. [25] reported that these mechanisms include a direct vagus nerve, which results in secretion of acetylcholine or catecholamines. Moreover, interaction with enteroendocrine cells can result in the production of numerous neuropeptides, intestinal hormones, neurotransmitters, or microbial-related molecular patterns [25]. Communication of the gut microbiome with the CNS is predominantly mediated through microbial-derived intermediates. The best described examples include SCFAs, secondary bile acids, and tryptophan metabolites, for example Bacillus-derived poly-gamma-glutamic acid (gamma-PGA), which is able to stimulate dendritic cells to the polarization of CD4^+^ cells toward Th1 and may also promote the differentiation of Treg cells [25]. Some of these compounds may directly interact with enteroendocrine cells, enterochromaffin cells, and the mucosal immune system, which significantly contributes to the ability to cross the intestinal barrier and enter circulation. This fact, in turn, allows for the crossing of the blood–brain barrier [26]. Additionally, the gut microbiome provides stimulating signals to the immune system that may activate innate and adaptive immune responses. Activation of the parasympathetic nervous system by the release of acetylcholine activates acetylcholine nicotinic receptor (α7nAChR), which protects against viral infections, including lowering macrophage inflammatory responses [27]. The adaptive immune system has also been shown to control the composition and diversity of the gut microbiome. Cytokine-mediated immune signaling from the gut to the brain is another proven communication pathway of the gut–brain axis. Gut bacteria are able to influence the synthesis of proinflammatory cytokines, such as TNF-α, IL-1β, IFN-γ, and IL-6, and anti-inflammatory cytokines, such as IL-10 [28]. The direct effect of the cytokines on the CNS is due to permeability in certain areas of the blood–brain barrier and the activation of afferent nerve fibers, e.g., the vagus nerve. Astrocytes are implicated in another notable interaction between the gut microbiome and the CNS. Astrocytes constitute the most abundant cell population in the CNS, participating in the control of the blood–brain barrier, regulation of the metabolism (glycogen storage), and support of neuronal signaling [29]. Microbial metabolites are able to activate aryl hydrocarbon receptors (AhRs) to decrease the inflammatory response by the regulation of type I interferon signaling in astrocytes [26,29]. These mechanisms are presented in Figure 1. The gut microbiota also regulate key central neurotransmitters, such as serotonin (via *Bifidobacterium infantis*), or the synthesis and release of neurotransmitters from γ-aminobutyric acid-synthesizing bacteria (*Lactobacillus and Bifidobacterium* spp.) [30].

The entry of the virus in the gut may lead to the migration of the virus to the CNS via the circulation or the vagus nerve. Glial cells and neurons have been shown to present receptors that mediate viral entry into the cells. To date, reports have indicated that a partial or complete loss of smell is an early marker of SARS-CoV-2 infection. The mechanisms responsible for smell dysfunction in COVID-19 are not well understood [31,32,33,34]; however, the possibility of subsequent brain infection should be considered due to the damage to the olfactory receptor neurons located in the olfactory epithelium, which contains the cells presenting protein receptors necessary for SARS-CoV-2 infection. Moreover, relatively high levels of ACE2 and transmembrane protease serine 2 expression were demonstrated in the olfactory mucosa of humans and mice. Smell dysfunction may also involve unidentified factors, according to Butowt and Bilinska [35], a cytokine storm caused by SARS-CoV-2 infection.

Mechanisms such as the direct vagus nerve, interaction with enteroendocrine cells and microbial-derived intermediates are involved in the interaction of the gut microbiota and central nervous system. Some of these significantly enable the intestinal barrier and blood–brain barrier to be crossed through increasing the permeability of those barriers due to the ability of the gut microbiota to influence the synthesis of cytokines. The gut microbiome stimulates the immune system to activate an innate and adaptive immune response and also may decrease the inflammatory response [25,26,36].

## 4. Lung Microbiome in SARS-CoV-2 Infection

*Fusobacterium, Haempophilus, Pervotella, Streptococcus*, and *Veillonella* are the main genera in the lungs of healthy individuals [13]. To identify the component of the lung microbiome potentially most relevant to the local host immune response, Segal et al. [37] analyzed correlations between cytokine profiles and viable communities of microorganisms. The authors reported that the presence of *Prevotella* and *Veillonella* positively correlated with the serum concentrations of a wide variety of cytokines, including Th17.

The lung microbiome is crucial for the host immune system, and the imbalance between microbial migration and removal predisposes the host to the progression and exacerbation of respiratory diseases [13,38].

The ACE2 receptor is present throughout the body, and the host tissues become potentially accessible to the infection during inhalation of SARS-CoV-2 due to the presence of a receptor that enables cellular entry of the virus with subsequent replication and invasion of new cells. If the immune system fails to resist SARS-CoV-2 at this initial stage, the virus moves to invade the lungs and multiply [13].

The lungs are an essential organ in the body’s defense system given a constant exposure to a variety of environmental stimuli [39]. An increasing amount of evidence indicates that the activation of pulmonary immune cells initiates innate and acquired immunity that requires exposure to microbes [39,40]. To date, only two studies have analyzed the lung microbiome of COVID-19 patients [39]. Shen et al. [41] investigated bronchoalveolar fluid in patients with COVID-19 and with non-nosocomial pneumonia and in healthy controls. The authors observed a significant alteration in the microbiota composition in the patients with COVID-19 and pneumonia compared to that in the healthy controls. The second study reported the characteristics of the lung microbiota in the autopsy of 20 deceased COVID-19 patients. Various types of pathogenic bacteria and fungi were identified. Significantly, the fungal species in the lung tissue that were usually dominated by *Cryptococcus* spp. infections caused by these microorganisms have high morbidity and mortality rates globally, especially in regard of immune suppression and CNS involvement [42].

The pulmonary microbiome remains poorly understood compared to the gut microbiome; however, currently reported observations confirm the key role of commensal lung microbiome in the immune tolerance that is maintained by alveolar macrophages and dendritic cell subpopulations [43]. These cell types induce Treg production [44] and produce prostaglandin E2, tumor growth factor-β (TGF-β), and IL-10 [39] to perform immunomodulatory functions.

**Figure 2:**
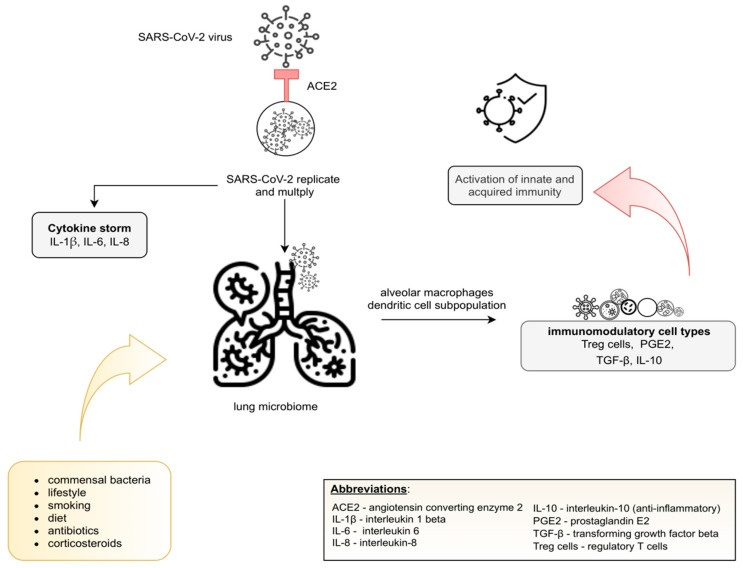
The lung microbiome after SARS-CoV-2 infection.

According to Conte et al. [45], numerous variables may influence the lung microbiota, including the composition of commensal bacteria and host immune response: lifestyle, diet, smoking, and use of certain drugs, including antibiotics and corticosteroids. A recent prospective study [46] showed that patients with a fiber-enriched diet better recover from COVID-19. It might be related to gut microbiota mediated systematic immunoregulation, through SCFA, which is also responsible for an anti-inflammatory response. Additionally, [47] has shown that the IFN-I signaling response is associated with avoiding excessive virus replication, inflammation and, thus, a cytokine storm. A potential mechanism of replication, multiplication and commensal lung microbiome is presented in Figure 2.

The ACE2 receptor enables cellular entry of the virus and moves to invade the lung, initiating inflammation, but also replication and multiplication. The pulmonary immune cells initiate an innate and acquired immunity response that requires exposure to the commensal lung microbiome. Immune tolerance is maintained by alveolar macrophages and dendritic cell subpopulations and, as a consequence of the production of immunosuppressive factors (Treg cells, PGE2, TGF-β, IL-10), by immunoregulatory cells. The composition of the lung microbiome may by influenced by the following variable factors: diet, lifestyle, use of certain drugs, including antibiotics and corticosteroids [13,43,44,45].

## 5. Gut–Lung Axis in COVID-19

The analysis of the molecular pathogenesis and diagnosis of COVID-10 by Li et al. [48] demonstrated that coronaviruses cause damage to the lung and lead to pneumonia with imbalanced hyperimmune responses [6]. Recent evidence demonstrated that bidirectional crosstalk between the gut and the lung is involved in the maintenance of immune homeostasis [49] and in disease pathways. In 2012, Keely et al. [50] demonstrated the effect of the gut microbiota on lung health through a bidirectional exchange between the gut microbiome and the lungs, which is defined as the gut–lung axis [6]. This link also involves the migration of immune cells from the gut to the respiratory tract through circulation, where these cells assist in the fight against an infection. Moreover, the gut regulates the pulmonary immune response through host-acquired inflammatory mediators detected in the serum. During viral respiratory infections, the macrophage response to the pathogens depends on the presence of gut microbes [13,51].

When endotoxins and metabolites of microbes that cause pulmonary inflammation enter the bloodstream, the composition of the gut microbiota may change [1,52], resulting in the enhancement of the relative abundance of *Bacteroidetes* and a reduction in the relative abundance of *Firmicutes* [53].

Only a few studies described the microbiome of patients suffering from COVID-19. Khatiwada et al. [39] investigated whether the pulmonary microbiome may influence SARS-CoV-2 infection and the outcomes of the disease and noted the lack of direct evidence linking the lung microbiome to the clinical course of COVID-19. However, the emerging evidence from human or animal investigations demonstrates that the lung microbial systems and gut microbiota is significantly altered in patients infected with SARS-CoV-2, which may influence the course and severity of COVID-19 symptoms [39,42,54,55]. A study compared the composition and diversity of the gut microbiome in patients with COVID-19 and patients with influenza A (H1N1). In the group of COVID-19 patients, a significantly reduced microbial diversity, a considerably higher relative abundance of opportunistic pathogens, such as *Streptococcus, Rothia, Veillonella*, and *Actinomyces*, and a lower relative abundance of symbiotic microorganisms were detected [55]. The authors concluded that the gut microbiome has potential diagnostic and therapeutic value in COVID-19; therefore, further studies are needed in this area. Moreover, the SARS-CoV-2 virus has also been detected in fecal samples of some infected patients [19,56]. The SARS-CoV-2 infection of gastrointestinal tissues was of interest to Hong Kong researchers who collected fecal samples of COVID-19 patients on hospital admission until discharge to investigate alterations in fecal microflora and its association with the severity of the disease and fecal shedding. The findings of this study suggested that patients with COVID-19 manifested a depleted gut microbiome compared to that in the controls, even after the removal of SARS-CoV-2 (measured by throat swabs) and resolution of the respiratory symptoms. The initial abundance of the *Firmicutes cluster, Coprobacillus genus*, and the *Clostridium hathewayi* species often correlated with COVID-19 severity (mild, moderate, severe, or critical) (Spearman correlation coefficient Rho >0.9, *p* <0.01). Anti-inflammatory bacteria *Faecalibacterium prausnitzi* and indole-positive bacteria *Alistipes onderdonkii* negatively correlated with the severity of COVID-19 (Spearman correlation coefficient Rho <−0.87, *p* <0.01 in the case of *F. prausnitzi*, Spearman correlation coefficient Rho <−0.09, *p* <0.005 for *A. onderdonkii*). Notably, these alterations were observed in all the COVID-19 patients who had not been treated with antibiotics before the infection [19]. Notably, *Actinomyces viscosus*, an opportunistic bacterium of the oral cavity and upper respiratory tract, was also identified in the gut of patients with COVID-19. Its presence suggests the passage or transfer of parenteral microbes into the gut. These data must be interpreted with caution because the study was carried out in a small group of 15 patients (the analysis of the relationships between alterations in the gut microbiota and the severity of COVID-19 was performed in a group of seven patients); therefore, the results require confirmation in a larger group. Similar results have been presented by Yeoh et al. [36], while investigating whether the gut microbiota is linked to severity in SARS-CoV-2-infected patients. They reported that any gut bacterial strains, *Faecalibacterium prausntizii, Eubacterium rectale,* and *Bifidobacteria*, known as immunomodulatory commensals, were under-presented in COVID-19 patients and remained decreased in stool samples 30 days after resolution.

On the other hand, Groves et al. [53] examined the associations between pulmonary viral infection on the gut microbiota and the gut environment using mouse models of infections with common respiratory pathogens, respiratory syncytial virus (RSV), and influenza virus. There was a significant change in the composition of the gut microbiome on days four and seven after RSV infection when compared to that on day zero (*p* = 0.006). The dominant phyla in all the mice before and after infection were *Bacteroidetes* and *Firmicutes*; however, after RSV infection, there was a significant increase in the relative abundance of *Bacteroidaceae* and a corresponding decrease in *Firmicutes*. Interestingly, the alterations in the microbiome and weight loss were transient. Already on day 14, the proportions of biodiversity of *Bacteroidetes* or *Firmicutes* phyla were similar to the parameters observed before the infection, thus demonstrating the resilience of the gut microbiota; 28 days after the RSV infection, the mice returned to their baseline weight. Thus, the authors concluded that infection with RSV changes the gut microbiome. Groves et al. [53] also noted significantly higher lipocalin-2 concentrations—a biomarker of intestinal inflammation—in the feces after RSV infection and also measured the levels of cytokines in the airways and colonic lavage after RSV infection. However, there was no detectable increase in the levels of IL-13 or IL-17 at either site, although IFN-γ was elevated in the airways after the RSV infection. Thus, the data of several studies demonstrated an increase in the fecal levels of calprotectin in patients suffering from COVID-19, which proves that SARS-CoV-2 infection also contributes to inflammatory reactions in the gut [20]. SARS-CoV-2 RNA was detected by Xiao et al. [57] in the fecal mucosa with infiltrates of plasma cells and lymphocytes with interstitial edema of the gastric, duodenal, and rectal epithelium. Moreover, the presence of the viral ACE2 receptor was demonstrated in the cytoplasm of gastrointestinal epithelial cells, and the viral nucleocapsid protein was detected in the cytoplasm of rectal, duodenal, and gastric epithelial cells.

Another pilot study of Zuo et al. [56] detected SARS-CoV-2 virus RNA in anal swabs and fecal samples of some COVID-19 patients, even in samples from the subjects negative for respiratory viral RNA. According to the authors, the transcriptional activity of viral infection and replication persists in the gut even after the removal of SARS-CoV-2 from the respiratory tract, thus suggesting implementation of a strategy to eliminate coronavirus activity in the gut by modulating the composition and functionality of the gut microbiome. However, the study could have been more relevant if it was not limited by a small group of respondents. Fecal samples were collected from only 15 hospitalized patients in China, and seven of them tested positive for SARS-CoV-2 on the first sampling based on metagenomic viral RNA sequencing.

Conversely, according to Pan et al. [10], cross-sectional studies conducted in the initial period of the pandemic (until March 2020) showed that viral RNA in fecal samples was detected in up to only 53.4% of the studied patients.

The presence of the SARS-CoV-2 virus in the feces of asymptomatic individuals indicates that COVID-19 may be transmitted through the feces [20,58]. On the other hand, studies reported by Zang et al. [59] showed that although the genomic RNA of SARS-CoV-2 was detected in fecal samples of COVID-19 patients, a virus suitable for reinfection was not detected. The authors primarily studied the replication ability of the virus in a mouse model and extended the scope of the study to include human samples. The results of the study showed that SARS-CoV-2 particles are able to enter enterocytes but cannot survive in the gastrointestinal tract due to the low pH of the gastric fluid and unfavorable intestinal environment where the virus contacts with bile and digestive enzymes [6,59]. The currently available results failed to demonstrate that SARS-CoV-2 virus in feces is a threat [6,59]; however, the impact of gut microbiota on the severity of COVID-19 should be investigated in further studies due to its bidirectional connection with the immune system [6].

**Figure 3:**
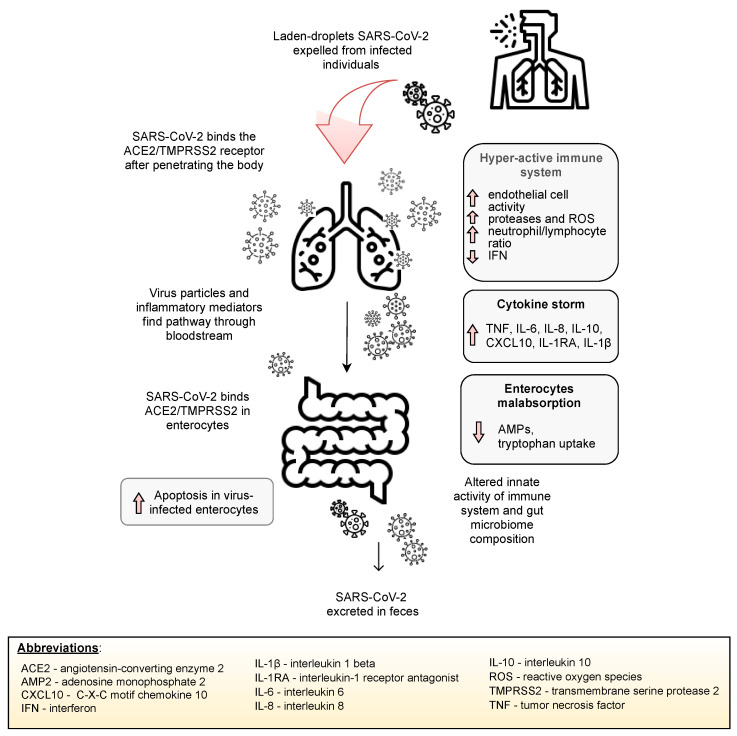
The potential mechanism of the involvement of the gut–lung axis in SARS-CoV-2 infection.

SARS-CoV-2 infection may lead to the lung hyper-permeability through increased inflammatory mediators on the grounds of infection, and therefore, the virus may migrate via circulation to the intestines. Inflammatory mediators present in circulation may also lead to an increase in the intestinal permeability, thus causing the leakage of gut microbiota metabolites and its entry via circulation to organs, such as the lungs [13]. Indeed, the gut microbiota and lungs appear to be closely linked. Figure 3 depicts a potential mechanism of the lung–gut axis in SARS-CoV-2 infection.

Inhalation of viral-laden droplets expelled from an infected individual may lead to the binding of SARS-CoV-2 molecules to the ACE2 enzyme and other receptors and entry into potential host cells. Stimulation of the host immune system leads to the release of inflammatory mediators and, consequently, may lead to a “cytokine storm”. Elevated concentrations of inflammatory mediators lead to the activation of endothelial cells and lung hyperpermeability; thus, SARS-CoV-2, along with inflammatory mediators, migrates to the gut via circulation and binds to ACE2 receptors expressed on enterocytes to influence the microbial structure and immune system. Inflammatory mediators disrupt the intestinal barrier, increasing gut permeability, resulting in the entry of microorganisms and their metabolites into circulation, enabling its migration to various organs, including the lungs [13,60].

## 6. Gut Microbiota and Immune System Functions

The gut microbiota, on top of its metabolic role, plays a key function in the development, instruction, and priming of the immune system [51]. Gut microflora signals tune immune cells to pro- and anti-inflammatory responses, thereby shaping susceptibility to a disease. Coordination of gut immune homeostasis is achieved by the induction of the development of proinflammatory responses, such as Th17 and regulatory T cells, that can produce immunoregulatory cytokines (e.g., IL-10, TGF-β, or IL-35) and, consequently, balance the mutual coexistence of microbial species. Moreover, Th17 cells and lymphoid tissue inducers induce IL-22 production and then decrease RegIIIy production to further transform the gut microbiome [61]. These processes were illustrated in a study of gnotobiotic (germ-free) mice, which presented a reduction in intestinal Tregs and gene expression of IL-10 compared to those in conventional mice [62]. Moreover, natural killer (NK) cells residing in nonmucosal lymphoid organs could not be effectively primed to elicit antiviral responses due to the lack of type 1 interferon production by macrophages and dendritic cells in germ-free mice [2].

The gut microbiota also play an important role in the production of IL-22 in the gut (formerly characterized as Th17 and Th22 cytokines), which is central to the host protection and induction of antimicrobial peptides and promotes protective functions of the epithelial barrier [63]. Innate lymphoid cells (ILC) and CD4^+^ T cells produce IL-22, although the factors regulating IL-22 production by CD4^+^ and ILC T cells are unknown. Recent reports indicate that microbiota-derived SCFAs promote the production of IL-22 by CD4^+^ and ILC T cells by inhibiting histone deacetylase and GPR41. SCFAs also elevate the production of IL-22 [62] by promoting the expression of the aryl hydrocarbon receptor and hypoxia-inducible factor 1α [63]. SCFAs, such as acetate, propionate, and butyrate, are the main energy sources for intestinal epithelial cells, promote the development of regulatory T cells, induce dendritic cells in the gut mucosa, and reduce autoimmunity. At the same time, microbial metabolites are integral for the promotion of the immune response against pathogens in the gut, including the induction of IL-18 or defensin secretion. Thus, the products of microbiota metabolism are important for an appropriate regulation of mucosal barrier integrity and immune homeostasis [51]. Violation of the mucin barrier facilitates the invasion of microbes through the epithelium and leads to inflammation [2].

People ≥65 years of age are more likely to die from COVID-19 than people <65 years of age. Age-related changes in the microbiota are very important [6]. The gut microbiota develop with age to increase diversity and stability. However, an opposite pattern is observed in the microbiome of elderly people [2,6]. A cohort study of 950 fecal samples (from which DNA was extracted in succession) [64] provided data on the composition of the gut microbiota of men over 65 years of age. A total of 275 mapped taxa of microbes were described indicating 30 types of microbes that dominated the microbiome of the male subjects. The most common bacterial strain was *Bacteroides* (31.2%, range: 0.02−97.86%), followed by *Faecalibacterium* (8.5%, 0.00−48,55%), *Alistipes, Akkermansia*, and *Prevotella*. *O’Toole and Jeffery* [65] reported another combined analysis evaluating the quantitate and qualitative alterations in microbiota based on the age of hosts that was carried out in a group of people 21 to 80 years of age; the results indicated that a decrease in the number of gut microbes is directly proportional to age and is associated with a simultaneous increase in microbial diversity. Similarly, a comparative analysis of the microbiota of centenarians versus the population of 70 years of age demonstrated significant differences in the microbiome between the groups. The microbiota of centenarians had a higher abundance of facultative anaerobes and a lower abundance of bacteria of the *Firmicutes phylum* and *Faecalibacterium prausnitzi*. Moreover, higher inflammation was observed in the group of centenarians [66]. The authors of another review [65] linked this observation to a reduced number of anti-inflammatory species with a considerably higher abundance in younger populations that maintain homeostasis. Gut microbiota dysbiosis caused by aging [13] and numerous comorbidities, including inflammation with reduced acquired immunity induced by B lymphocytes, impairment of the interferon type I pathway of plasmacytoid dendritic cells, and decreased expression of ACE2, may result in high mortality in elderly patients infected with the SARS-CoV-2 virus [67].

Antibiotic treatment during SARS-CoV-2 infection may be another reason for alteration in the gut microbiota. A large number of patients in China were given antibiotics (58–71% of patients) during COVID-19 treatment [7,68]. Antibiotics may lead to intestinal dysbiosis and increase susceptibility to new infections and inflammation. Additionally, antibiotics may cause antibiotic-related diarrhea.

To date, there is no clear evidence as to whether gut microbiota dysbiosis is one of the factors determining the severity of COVID-19 disease or, on the contrary, whether viral infection modulates unfavorable alterations in the gut microbiome.

SARS-CoV-2 may infect people of any age; however, elderly people and people with comorbidities are more susceptible to complications and have a higher risk of mortality due to the infection [20]. Smoking, male sex, and comorbidities, especially cardiovascular diseases, hypertension, diabetes [69], Alzheimer’s disease, and dementia [20], play an essential role in defining the risk of the development of COVID-19 disease. SARS-CoV-2 infection predisposes patients to an increased risk of cardiovascular complications, including myocardial infarction, arrhythmia, stroke, or myocardial depression. The decreased diversity of the microbiota accompanying cardiovascular diseases [70] significantly worsens the prognosis of COVID-19 disease [20]. Importantly, obesity is also associated with alterations in the gut microbiota and is another highly significant risk factor in COVID-19. Adipose tissue can serve as a reservoir for the SARS-CoV-2 virus. The adipocytes of obese patients show higher concentrations of ACE2; therefore, adipose tissue may contribute to the spreading of SARS-CoV-2. Moreover, disturbance of the cytokine profile in obese patients (increased concentrations of proinflammatory adipokines, leukotrienes, and chemerin) may result in an elevated risk of death in response to COVID-19 infection [20]. In some patients, type 2 diabetes mellitus may be accompanied by a decreased expression of the ACE2 enzyme. Several mechanisms are responsible for poor prognosis in diabetic patients with COVID-19; however, the influence of the pharmaceuticals used for the treatment of SARS-CoV-2 infection (corticosteroids, lopinavir, and ritonavir) may contribute to the deterioration of glycemic control in people with type 2 diabetes mellitus and, thus, induce additional impairment of the innate immune response, promote the production of proinflammatory cytokines, and intensify a decrease in the expression of ACE2 [71].

Sharma et al. [72] investigated the potential role of metformin in the treatment of COVID-19. The authors noted that metformin was originally introduced as an anti-influenza drug, and the glucose-lowering effect of metformin is only one of its side effects. The possible therapeutic effect of metformin in SARS-CoV-2 infection is due to the activation of the protein kinase activated by AMP (AMPK) in hepatocytes, which leads to the phosphorylation of ACE2 and its conformational and functional changes. Ultimately, this may cause a subsequent increase in ACE2 expression via AMPK that may lead to a decrease in SARS-CoV-2 binding [72]. The upregulation of ACE2 expression by metformin not only prevents droplets of SARS-CoV-2 from entering the body but also protects against ACE2 activation via AMPK signaling. In addition to AMPK activation, metformin has also been shown to inhibit the rapamycin pathway (mTOR), which plays an important role in the pathogenesis of influenza. Cell signaling networks, which include mTOR, were reported to play a significant role in the pathogenesis of infection induced by MERS-CoV-2, which is similar to SARS-CoV-2 [73]. The prevention of viral replication due to the inhibition of mTOR signaling is another potential mechanism of the therapeutic role of metformin. Metformin has been shown to be a direct modulator of the mTOR complex [66], and the role of mTOR was confirmed by the molecular analysis focused on mapping SARS-CoV-2 interactions with human proteins reported by Gordon et al. [74].

Limited in vivo and in vitro studies have shown that metformin may inhibit hepatitis C virus. Chan et al. [75] reported a decrease in the antiviral response due to impaired insulin sensitivity. The authors concluded that improving insulin sensitivity could supplement antiviral therapy in patients infected with hepatitis C virus. Another study investigated the effects of rosiglitazone and metformin on inflammation and glycemia (fasting and postprandial) in HIV-infected patients with lipodystrophy [76]. A significantly reduced HOMA index and decreased concentration of monocyte chemoattractant protein 1 were observed. The results of the study indicated a potential protective effect of metformin and the prevention of atherosclerosis [75]. The role of metformin in the inhibition of the viruses appears to be promising, and further clinical trials are needed.

Proton pump inhibitors (PPIs) are acid-suppressive agents and are among the most widely used and overused drugs in the world [77], e.g., in GERD treatment [78]. Many studies demonstrated that the long-term use of PPIs exposes patients to an elevated risk of developing extraintestinal disorders due to PPI-driven gastric hypochlorhydria that consequently leads to alterations in the microbiome composition in all regions of the gastrointestinal tract [77]. Recent attention was focused on the changes in the gut microbiome associated with the use of PPIs [77,78,79] and in correlation with SARS-CoV-2 infection [80,81,82]. Imhan et al. [79] demonstrated that the gut microbiome plays an important role in enteric infections. Analysis of the gut microbiome composition of 1815 individuals spanning three cohorts indicated that PPI use is associated with decreased bacterial richness and profound alterations in the microbiota, which is even more pronounced than the changes induced by the use of antibiotics or other drugs. The reviewed evidence apparently suggests a pertinent role for gut microbiota in the resistance and promotion of the microbial colonization of the gut by *Clostridium difficile* and other enteric infections via several mechanisms that either inhibit bacterial growth or enhance the immune system [79] and result in an increased risk of pneumonia due to the effects on the activities of polymorphonuclear neutrophils, cytotoxic T cells, and NK cells [80].

Current studies also investigate the potential associations of the current use of PPIs with the infection rate of COVID-19 in patients tested for SARS-CoV-2. The Korean nationwide cohort included 132,316 patients, and 14,163 of them were current PPI users; the use of PPIs was associated with a 79% increase in the risk of severe clinical outcomes of COVID-19, and the relationship with the past use of PPIs was insignificant. Moreover, this study demonstrated that current PPI use within the past 30 days was associated with a 90% increase in the risk of severe clinical outcomes of COVID-19. Conversely, there was no evidence that the use of PPIs increases susceptibility to SARS-CoV-2 infection [80], which is important for clinicians [80,82]. A possible explanation of this association suggests that individuals with higher virus colonization in the stomach (due to elevated gastric alkalinity caused by PPI administration) may be more susceptible to a severe course of SARS-CoV-2 infection. Other studies that investigated the impact of the use of PPIs on microbiota and COVID-19 severity demonstrated that the use of PPIs or famotidine (another medication for gastric diseases) is associated with a higher risk of severe COVID-19 disease in a Chinese cohort [81]. A beta-analysis study by Kow and Hasan [82] included five studies published after September 2020 in a total of 37,372 patients; the results indicated that the use of PPIs not only increased the odds for a severe course of COVID-19 but also increased the odds for the development of secondary infections compared to patients who did not use PPIs. These findings suggest that clinicians should be aware of the influence of PPIs on the microbiome and its relationship with COVID-19 disease [82].

## 7. Conclusions

Numerous studies confirmed the key role of the microbiota in the activation of the response of the innate and adaptive immune systems against viral infections. Discovery of quantitative and qualitative changes in the microbiota composition and their metabolic products in the context of COVID-19 can help to identify new disease biomarkers and new therapeutic targets.

While there is no clinical evidence that targeting the microbiota and lung–gut axis is an effective therapeutic strategy for SARS-CoV-2 infection, the use of probiotics, prebiotics, and a high-fiber diet may reduce inflammation, maintain gut biodiversity, and support the immune system.
